# Supplementary material for: Genomic analysis of Acinetobacter baumannii DUEMBL6 reveals diesel bioremediation potential and biosafety concerns
Source: PLoS One. 2026 Jan 20;21(1):e0339456. doi: 10.1371/journal.pone.0339456 (PMC12818619; doi:10.1371/journal.pone.0339456)
Supplement: S1 Table — (PDF) [file pone.0339456.s001.pdf]

S1 Table. List of antimicrobial resistance genes detected in DUEMBL6.

Antimicrobial Resistance Genes	Database	Identity	Coverage	Drug Class	Resistance Mechanism
blaOXA-338	resfinder, card	98.79	100	BETA-LACTAM	Antibiotic inactivation
blaOXA-781	ncbi	99.39	100	BETA-LACTAM	Antibiotic inactivation
blaOXA-800	argannot	99.03	100	BETA-LACTAM	Antibiotic inactivation
blaADC-25	resfinder	97.14	100	CEPHALOSPORIN	Antibiotic inactivation
blaADC-2	argannot	99.65	100	CEPHALOSPORIN	Antibiotic inactivation
blaADC-158	ncbi	100	100	CEPHALOSPORIN	Antibiotic inactivation
blaA1	Megare, argannot	93.9	99.61	cephalosporins, carbapenems	Antibiotic inactivation
ant(3")-IIa	Ncbi, megares, card	98.86	100	SPECTINOMYCIN;STREPTOMYCIN	Antibiotic inactivation
ADET2	megares	98.19	100	glycylcycline;tetracycline	Antibiotic efflux
ADET1	megares	97.81	99.78	glycylcycline;tetracycline	Antibiotic efflux
adeS	Megares, card	96.24	98.07	glycylcycline;tetracycline	Antibiotic efflux
adeR	Megares, card	97.98	100	glycylcycline;tetracycline	Antibiotic efflux
adeA	Megares, card	98.57	100	glycylcycline;tetracycline	Antibiotic efflux
adeB	Megares, card	98.33	100	glycylcycline;tetracycline	Antibiotic efflux
abeS	megares,	99.39	100	aminocoumarin;macrolide	Antibiotic

	card				efflux
abeM	megares, card	99.41	100	acridine_dye;fluoroquinolone;triclosan	Antibiotic efflux
adeK	megares, card	99.04	100	fluoroquinolone;tetracycline	Antibiotic efflux
adeJ	megares, card	99.62	100	fluoroquinolone;tetracycline	Antibiotic efflux
adeI	megares, card	99.92	100	fluoroquinolone;tetracycline	Antibiotic efflux
adeH	megares, card	98.48	100	fluoroquinolone;tetracycline	Antibiotic efflux
adeG	megares, card	97.83	100	fluoroquinolone;tetracycline	Antibiotic efflux
adeF	megares, card	98.94	100	fluoroquinolone;tetracycline	Antibiotic efflux
adeL	megares, card	99.31	100	fluoroquinolone;tetracycline	Antibiotic efflux
mexT	megares	98.79	100	Wide range	Antibiotic efflux
abaQ	megares, card	97.93	100	quinolones	quorum-sensing
amvA	megares, card	98.85	100	acridine_dye;macrolide	Antibiotic inactivation
