# Supplementary material for: Genomic analysis of Acinetobacter baumannii DUEMBL6 reveals diesel bioremediation potential and biosafety concerns
Source: PLoS One. 2026 Jan 20;21(1):e0339456. doi: 10.1371/journal.pone.0339456 (PMC12818619; doi:10.1371/journal.pone.0339456)
Supplement: S2 Table — (PDF) [file pone.0339456.s002.pdf]

**S2 Table. List of virulence genes detected in DUEMBL6.**

| Virulence genes | Identity (%) | Functional annotations |
|---|---|---|
| *csuE* | 96.37 | Csu pilus tip adhesin CsuE |
| *csuD* | 97.84 | Csu pilus usher protein CsuD |
| *csuC* | 98.56 | Csu pilus chaperone protein CsuC |
| *csuB* | 99.81 | Csu pilus subunit CsuB |
| *csuA* | 96.64 | Csu pilus subunit CsuA |
| *csuA/B* | 100.00 | Csu pilus major pilin subunit CsuA/B |
| *plcD* | 99.32 | phosphatidylserine/phosphatidylglycerophosphate/cardiolipin synthase |
| *plc* | 97.17 | phospholipase C |
| *plc* | 97.42 | phospholipase C |
| *pgaD* | 98.28 | poly-beta-1,6-N-acetyl-D-glucosamine biosynthesis protein PgaD |
| *pgaC* | 98.32 | poly-beta-1,6 N-acetyl-D-glucosamine synthase |
| *pgaB* | 98.09 | poly-beta-1,6-N-acetyl-D-glucosamine N-deacetylase PgaB |
| *pgaA* | 98.54 | poly-beta-1,6 N-acetyl-D-glucosamine export porin PgaA |
| *bauF* | 98.72 | siderophore-interacting protein |
| *basJ* | 98.29 | acinetobactin biosynthesis protein BasJ |
| *bfmR* | 99.58 | biofilm-controlling response regulator |
| *bfmS* | 98.00 | signal transduction histidine kinase |
| *basA* | 95.51 | acinetobactin biosynthesis protein |
| *basB* | 95.65 | non-ribosomal peptide synthetase with condensation and peptidyl carrier protein domains |
| *bauD* | 96.23 | ferric siderophore ABC transporter, permease protein |

| | | |
|---|---|---|
| | | BauD |
| *bauC* | 97.15 | ferric siderophore ABC transporter, permease protein BauC |
| *bauE* | 98.57 | ferric siderophore ABC transporter, ATP-binding protein BauE |
| *bauB* | 98.35 | ferric siderophore ABC transporter, periplasmic siderophore-binding protein |
| *basC* | 98.32 | acinetobactin biosynthesis protein BasC |
| *basD* | 97.26 | acinetobactin biosynthesis protein BasD |
| *basF* | 97.47 | aryl carrier protein BasF |
| *basG* | 97.31 | acinetobactin biosynthesis protein BasF |
| *basH* | 97.55 | non-ribosomal peptide biosynthesis thioesterase BasH |
| *barA* | 96.40 | siderophore efflux system of the ABC superfamily |
| *barB* | 97.93 | siderophore efflux system of the ABC superfamily |
| *entE* | 96.99 | non-ribosomal peptide synthetase adenylate-forming enzyme of acinetobactin synthesis |
| *omp A* | 99.25 | outer membrane protein OmpA |
| *abaR* | 95.12 | DNA-binding HTH domain-containing protein |
