# Supplementary material for: Genomic analysis of Acinetobacter baumannii DUEMBL6 reveals diesel bioremediation potential and biosafety concerns
Source: PLoS One. 2026 Jan 20;21(1):e0339456. doi: 10.1371/journal.pone.0339456 (PMC12818619; doi:10.1371/journal.pone.0339456)
Supplement: Attachment — Submitted filename: Response_to_Reviwer.docx [file pone.0339456.s003.docx]

1. Additional Editor Comments:

   Thank you for your submission. Upon reviewing the manuscript, we found that Figures 1 to 7 are not sufficiently clear in their current form. To proceed with the review process, we kindly request that you resubmit the manuscript with high-resolution versions of these figures to ensure their clarity and legibility for the reviewers.

The previous lower resolution may have resulted from the conversion to a combined PDF during initial submission. According to the journal, ‘**The compiled submission PDF includes low-resolution preview images of the figures after the reference list. The function of these previews is to allow you to download the entire submission as quickly as possible. Click the link at the top of each preview page to download a high-resolution version of each figure**.’ Yet, we have replaced some of the figures with high resolution versions.
