# Supplementary material for: Genomic analysis of Acinetobacter baumannii DUEMBL6 reveals diesel bioremediation potential and biosafety concerns
Source: PLoS One. 2026 Jan 20;21(1):e0339456. doi: 10.1371/journal.pone.0339456 (PMC12818619; doi:10.1371/journal.pone.0339456)
Supplement: Attachment — Submitted filename: Response_to_Reviwer_auresp_2.docx [file pone.0339456.s004.docx]

****REVIEWER 1****

1. The paper would benefit from further background discussion of the alkB and catE genes, their roles biodegradation, and previous uses in the field.

**Response to Comment 1:
We thank the reviewer for this insightful suggestion. In response, we have expanded the discussion of the alkB and catE genes to include both their established roles in hydrocarbon biodegradation and their previous applications in the field. Specifically, we now highlight that alkB-encoded alkane hydroxylases are widely used as biomarkers for alkane degradation in marine and soil environments, while catE-encoded catechol-2,3-dioxygenases play a central role in aromatic compound degradation and have been applied in the bioremediation of oil-contaminated soils and groundwater (van Beilen & Funhoff, 2007; Harayama et al., 1999; Wang et al., 2011; Jiao et al., 2016). These additions are included in the Results and Discussion section (Lines 251-255).**

2. **The authors mention comparison of % diesel degradation to other previously tested strains (lines 269-270), but these analyses were not done “side by side” as far as I can see. Were similar conditions used, so as to make this a valid comparison? Ideally, it would have been optimal to include other control/comparison strains in their analysis.**

**Response to Comment 2:**

**We thank the reviewer for this valuable comment. Since different studies were conducted under varying experimental conditions, we have included the respective conditions in Table 3 for proper context. It was not possible to identify studies performed under exactly identical parameters, as a study may have preferential temperatures and conditions based on their region or specific goals. Therefore, the comparisons presented here are intended to provide contextual rather than experimental insights into the relative degradation efficiency of our isolates. This clarification has been added to the revised manuscript (Lines 260–266, Table 3).**

3. **I did not see any figure legends in the material that was available for review. Please provide figure legends for the reader.**

**Response to comment 3**

We thank the reviewer for this suggestion. Figure legends have now been added to accompany all figures to provide clarity for the reader.

**4.** The authors discuss genes/relevant pathways that are present in the sequenced Acinetobacter isolate. The study would benefit from deletion/inactivation of some of these genes and then observing if this affects the biodegradation activity.
**5.** The authors discuss the presence of genes/systems for biosynthesis pathways in the sequenced isolate (as opposed to biodegradation). Is there any evidence that these pathways are functional? Could some assays be performed to test this?
**6.** The authors discuss the fact that virulence genes and antibiotic resistance genes were detected in the sequenced Acinetobacter isolate and that these genes could pose a problem for the use of the strain in biodegradation. The authors should suggest that these genes could possibly be deleted/inactivated in this strain, and then the resulting strain tested to see if this affects the biodegradation activity. In other words, the strain could possibly be engineered to eliminate virulence and antibiotic resistance for future use/applications. Experiments toward this end would improve the paper.

**Response to comments 4, 5, and 6:**
We thank the reviewer for these insightful suggestions. The primary scope of the present study was to identify and characterize the isolate and to provide a genomic overview of its biodegradation potential. We agree that functional validation through gene deletion/inactivation studies (comment 4), biochemical assays to test the activity of biosynthetic pathways (comment 5), and engineering approaches to remove virulence and antibiotic resistance determinants (comment 6) would substantially strengthen the understanding and potential application of this strain. We have now acknowledged these points in the Conclusion (lines 503-508) as **important directions for future work**. At present, we are seeking **funding support** to pursue these experimental approaches, and we believe that the publication of **this study will help us justify and build the foundation for such follow-up research**.

7. Was the sequenced isolate tested for relevant antibiotic resistance? This would improve the paper and give insight into whether those genes are expressed/functional.

**Response to comment 7**

We thank the reviewer for this valuable suggestion. We had previously performed phenotypic antibiotic resistance testing, but initially excluded these results to keep the focus on genomic characteristics and avoid excessive length. As suggested, we have now incorporated these data and linked the genomic resistance determinants to the corresponding phenotypic profiles (Lines 198-204, 419-421, 424-426, 434-436, and 442-443).

8. Whenever the authors refer to the sequenced strain DUEMBL6, it would help the reader if they include the genus/species in key locations to help the reader (some examples: titles to Figures 4, 5, and 7; line 357, line 362, etc). I realize you mention this strain in the text, but it helps the reader overall when reading the whole paper to include the genus/species, especially since there are many strain numbers used in the paper (for example, write as “Acinetobacter baumannii DUEMBL6”).

**Response to comment 8**

We thank the reviewer for this valuable suggestion. We have revised the manuscript to include the genus and species name (e.g., Acinetobacter baumannii DUEMBL6) in the specified figure titles (Figures 4, 5, and 7) and key text locations (lines 357, 362, etc.). We believe this change improves clarity and consistency for readers, especially given the presence of multiple strain numbers in the manuscript.

****REVIEWER 3****

1. Comparative Performance of DUEMBL6
   o Please consider including a comparative framework, such as a table summarizing the diesel degradation efficiencies of DUEMBL6 alongside those of other globally reported strains or consortia. This will help contextualize your findings.

**Response to Comment 1:
We thank the reviewer for this thoughtful suggestion. In response, we have included a comparative framework (Table 3) summarizing the diesel degradation efficiencies of A. baumannii DUEMBL6 alongside other globally reported strains and consortia. Since these studies were conducted under different environmental and experimental conditions, we have also included their respective parameters (e.g., temperature, incubation period) to ensure proper contextualization. The comparison is intended to provide perspective on the relative degradation efficiency of DUEMBL6 rather than a direct experimental equivalence. These additions can be found in the revised manuscript (Lines 260–266, Table 3).**

2. Biosafety and Application Risks
   The biosafety discussion requires more depth. I recommend elaborating on potential strategies such as the use of non-pathogenic microbial consortia, genetic attenuation of virulence factors, and immobilization or encapsulation techniques to prevent uncontrolled spread.

**Response to comment 2**

We thank the reviewer for this important suggestion. We agree that the biosafety aspects of applying *A. baumannii* DUEMBL6 in bioremediation require more depth. We have now expanded the Discussion to highlight potential strategies to mitigate these risks, including the use of non-pathogenic microbial consortia, genetic attenuation of virulence and resistance factors, and immobilization or encapsulation techniques to prevent uncontrolled environmental spread (Lines 490–495). We believe this addition strengthens the manuscript by addressing biosafety considerations and providing a framework for future applied research.

3. Functional Validation of Genomic Insights
The alkB mutation analysis (I138F, A334T) is interesting, but conclusions regarding functional advantage remain speculative. Please acknowledge this as a limitation and recommend future work, such as enzyme activity assays, transcriptomics, and proteomics, to validate functionality.

**Response to comment 3**

We thank the reviewer for this valuable suggestion. We have acknowledged the speculative nature of the alkB mutation analysis as a limitation and have recommended future validation through enzyme activity assays, transcriptomics, and proteomics in the Conclusion (Lines 501–505).

4. Figures and Visuals
A summary table of antibiotic resistance genes and virulence factors should be moved into the main text for ease of reference rather than being confined to supplementary materials.

**Response to comment 4**

We appreciate the reviewer’s suggestion. However, the summary tables of antibiotic resistance genes and virulence factors span more than one page, and per the journal’s formatting requirements, such large tables must remain in the supplementary materials. Therefore, we have kept these tables in the supplementary file.
5. Language and Formatting
o The manuscript would benefit from polishing for clarity and consistency. In particular, I noted several typographical errors that should be corrected:
a) Page 31, Line 306: “Idetification of biosynthetic gene clusters” → “Identification of biosynthetic gene clusters”
b) Page 36, Line 366: “Antibitotic resistance genes and mobile genetic elements” → “Antibiotic resistance genes and mobile genetic elements”
c) Page 20, Line 219 / Table 2: “Strenotrophomonas maltophila” → “Stenotrophomonas maltophilia”

**Response to comment 5**

We thank the reviewer for pointing out these errors. The typographical and formatting issues noted (Pages 20, 31, and 36) have been corrected in the revised manuscript.
