# Supplementary material for: Genomic analysis of Acinetobacter baumannii DUEMBL6 reveals diesel bioremediation potential and biosafety concerns
Source: PLoS One. 2026 Jan 20;21(1):e0339456. doi: 10.1371/journal.pone.0339456 (PMC12818619; doi:10.1371/journal.pone.0339456)
Supplement: Attachment — Submitted filename: Response_to_Reviwer_auresp_3.docx [file pone.0339456.s005.docx]

**As per Reviewer 2 comments, the manuscript discusses virulence and antibiotic resistance genes but lacks experimental validation of their functional roles. The authors were encouraged to consider gene deletion or inactivation studies targeting key virulence and antibiotic resistance determinants in future work. Such experiments would offer valuable insights into the genetic basis of resistance and virulence and could demonstrate the feasibility of engineering strains with reduced pathogenicity and resistance. However, it is understandable that these experiments may not be feasible within the current revision timeline.**

**Response to Comment 1**

**We thank the reviewer for this constructive comment. We agree that functional validation through gene deletion or inactivation studies would provide deeper insights into the roles of key virulence and antibiotic resistance determinants. While these experiments are beyond the scope of the current revision, we previously added this point to the conclusion as a prospect for future work. We believe this addition not only acknowledges the limitation but also highlights a clear direction for extending the findings of this study.**

**Reviewer 2 also noted that the authors had mentioned performing antibiotic resistance assays in the previous revision. Upon review, it was observed that while the assay has been described in the Methods section and discussed in the Results, the corresponding data (e.g., figures, tables, or supplementary material) are missing. Therefore, it is recommended to revise the manuscript and incorporate the missing antibiotic resistance assay data (figures or tables) to support their discussion in the manuscript.**

**Response to Comment 2
We thank the reviewer for pointing this out. We have now incorporated the missing antibiotic resistance assay data in Table 5 (Line 428) to support the corresponding descriptions in the Methods and Results sections. This table summarizes the phenotypic susceptibility profile.**
